# Gene Expression Profile as a Predictor of Seizure Liability

**DOI:** 10.3390/ijms24044116

**Published:** 2023-02-18

**Authors:** Anssi Lipponen, Natallie Kajevu, Teemu Natunen, Robert Ciszek, Noora Puhakka, Mikko Hiltunen, Asla Pitkänen

**Affiliations:** 1 A. I. Virtanen Institute for Molecular Sciences, University of Eastern Finland, P.O. Box 1627, FIN-70211 Kuopio, Finland; 2 Expert Microbiology Unit, Finnish Institute for Health and Welfare, P.O. Box 95, FIN-70701 Kuopio, Finland; 3 Institute of Biomedicine, University of Eastern Finland, P.O. Box 1627, FIN-70211 Kuopio, Finland

**Keywords:** attrition, cell culture, drug, gene expression, ictogenicity, neuronal

## Abstract

Analysis platforms to predict drug-induced seizure liability at an early phase of drug development would improve safety and reduce attrition and the high cost of drug development. We hypothesized that a drug-induced in vitro transcriptomics signature predicts its ictogenicity. We exposed rat cortical neuronal cultures to non-toxic concentrations of 34 compounds for 24 h; 11 were known to be ictogenic (tool compounds), 13 were associated with a high number of seizure-related adverse event reports in the clinical FDA Adverse Event Reporting System (FAERS) database and systematic literature search (FAERS-positive compounds), and 10 were known to be non-ictogenic (FAERS-negative compounds). The drug-induced gene expression profile was assessed from RNA-sequencing data. Transcriptomics profiles induced by the tool, FAERS-positive and FAERS-negative compounds, were compared using bioinformatics and machine learning. Of the 13 FAERS-positive compounds, 11 induced significant differential gene expression; 10 of the 11 showed an overall high similarity to the profile of at least one tool compound, correctly predicting the ictogenicity. Alikeness-% based on the number of the same differentially expressed genes correctly categorized 85%, the Gene Set Enrichment Analysis score correctly categorized 73%, and the machine-learning approach correctly categorized 91% of the FAERS-positive compounds with reported seizure liability currently in clinical use. Our data suggest that the drug-induced gene expression profile could be used as a predictive biomarker for seizure liability.

## 1. Introduction

Seizures related to drug use are severe adverse reactions that can prevent the drug from entering the market or lead to its withdrawal [1,2]. According to recent analysis, the cost of developing a new drug and achieving marketing approval is approximately $1.2 billion [3]. Therefore, the early detection of factors that might decrease the risk of later attrition would have a major influence on drug development.

Several in vitro and in vivo analysis platforms with medium throughput have been used for the early detection of the seizure liability (ictogenicity) of novel drug candidates [4]. These platforms include brain slice and multi-electrode array analysis of drug-induced epileptiform activity in rodent cortical neurons or slices, or human induced pluripotent cells [5,6,7,8,9,10,11,12,13,14], and the analysis of motor behavior generated by zebrafish larvae exposed to convulsant drugs [15,16]. Despite the promising data and increasingly stronger predictive values achieved by applying machine-learning analysis approaches, the predictive accuracy remains limited or has not yet been vigorously validated in different laboratories [17]. 

Recent in vivo analyses of the brain tissue from rodent models of epilepsy or from surgically removed human epileptic tissue revealed aberrant transcriptional regulation, acquired channelopathies, acquired synaptopathies, and neuroinflammation as mechanisms contributing to seizure generation [18,19]. Similarly, seizures induced in vivo by seizure-inducing drugs, such as pentylenetetrazol [20] and kainic acid [21], or in vitro by bicuculline [22] and (S)-AMPA [23], induce remarkable differential post-seizure/exposure gene expression. These findings support the idea that a drug-induced gene expression signature could be used as an indicator of the seizure-related attrition risk at an early phase of drug development.

Because seizures induce neuronal transcriptional changes as downstream cellular events, we hypothesized that the regulation of a specific set of mRNAs in neuronal cell cultures induced by a compound in vitro would report on its seizure-inducing ability in vivo. We exposed rat cortical neuronal cell cultures to non-toxic concentrations of 34 compounds for 24 h, 11 of which are known to induce seizures in vivo (tool compounds), 13 of which are associated with a high number of seizures in the FDA Adverse Event Reporting System (FAERS) or in the literature (FAERS-positive compounds), and 10 of which have no/a very low number of reported seizure-related adverse events (FAERS-negative). The compound-induced neuronal transcriptomics changes were measured from the mRNA-sequencing data. Bioinformatics and machine learning were used to define the in silico molecular fingerprint of each compound. Finally, the alikeness of the transcriptomics signature of a given FAERS-positive test compound with that of any of the known seizure-inducing tool compounds was scored, and eventually used to determine the risk of the compound’s ictogenicity.

## 2. Results

To define the ictogenetic gene expression signature, we used 11 tool, 13 FAERS-positive, and 10 FAERS-negative compounds. The tool compounds are known to induce seizures in animal models, and some also in humans [24,25,26,27]. The FAERS-positive compounds are associated with frequent seizures, and the FAERS-negative compounds are associated with no or a very low number of “seizures” reported as adverse events in the FDA Adverse Event Reporting System database (FAERS) [28] and the WHO VigiAccess database [29].

### 2.1. Highest Tolerated Concentration (Cytotoxicity)

**Figure 1:**
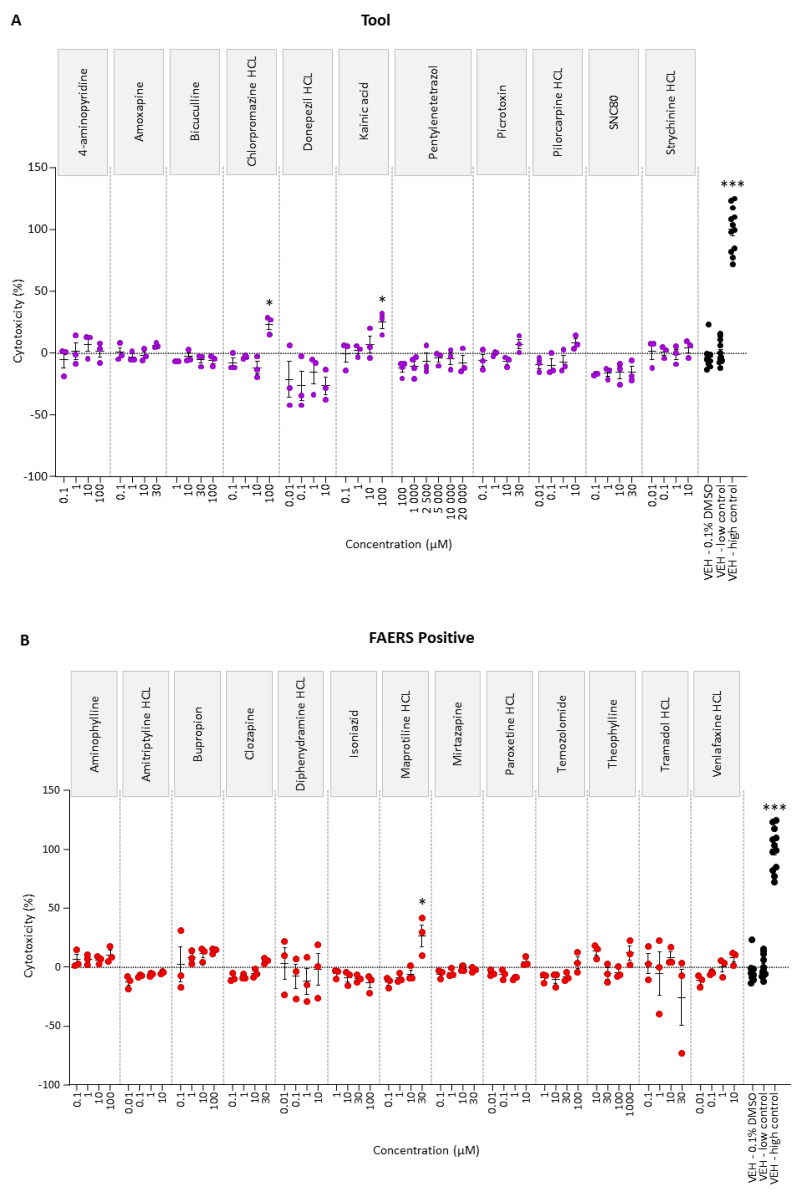

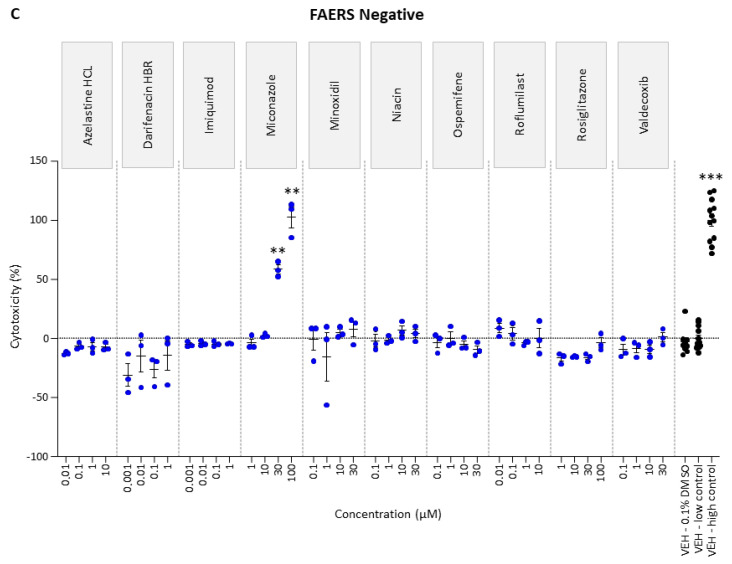
Cytotoxicity analysis of (**A**) tool, (**B**) FAERS-positive, and (**C**) FAERS-negative compounds in rat cortical neuronal cell cultures. Each compound was tested using four concentrations and pentylenetetrazol using six concentrations. ***Abbreviations***: VEH, vehicle. ***Statistical significance***: * *p* < 0.05, ** *p* < 0.01, *** *p* < 0.001 (vehicle 0.1% DMSO vs. compound-treated sample, Kruskal–Wallis followed by Mann–Whitney U-test). Data are presented as mean ± SEM.

To determine the highest tolerated dose, rat cortical neuronal cultures were exposed to four concentrations of each of the tool, FAERS-positive, and FAERS-negative compounds. Of the 34 compounds, only kainic acid (30 µM), chlorpromazine (100 µM), maprotiline (30 µM), and miconazole (30 and 100 µM) induced cytotoxicity (Figure 1). 

### 2.2. Number of Differentially Expressed (DE) Genes in Rat Cortical Neuronal Cell Cultures Is Dose-Dependent

First, we performed a dose-optimization study to demonstrate that a 24-h exposure of rat primary cortical cell cultures to various compounds will induce gene expression at non-toxic doses. Of the five compounds studied, four (all but aminophylline) induced a dose-dependent increase in the number of DE genes when both the false discovery rate (FDR) < 0.05 and the log2 transform of the absolute value of the fold-change (FC) > 1.5 were used as criteria (Table 1). The greatest dose-dependency was found after exposure of the cultures to pentylenetetrazol, as 1000 µM did not induce any DE genes whereas 20,000 µM induced 851 DE genes. 

### 2.3. All Compounds, except Paroxetine, Induced Alterations in Gene Expression in Rat Cortical Neuronal Cultures

Next, we increased the depth of the sequencing from 15–18 M reads to 27–33 M and the read length from 2 × 50 bp to 2 × 100 bp. All the compounds, except paroxetine, induced differential gene expression (FDR < 0.05 and log2(|FC|) > 1.5) in the rat primary cortical neuronal cell cultures after 24-h incubation (Table 2).

Of the 10 tool compounds, the highest number of DE genes was induced by amoxapine (2659), followed by SNC80 (2308) and pentylenetetrazol (1014). The numbers of DE genes induced by the remaining tool compounds ranged from 21 to 1014. 

Of the 13 FAERS-positive compounds, the highest number of DE genes (3695) was induced by clozapine. The number of DE genes induced by the remaining FAERS-positive compounds ranged from 3 to 827. 

Of the 11 FAERS-negative compounds, the highest number of DE genes was induced by imiquimod (2214), then rosiglitazone (1827). The number of DE genes induced by the remaining FAERS-negative compounds ranged from 7 to 1686. 

### 2.4. Similarities in Gene Expression between the Tool, FAERS-Positive, and FAERS-Negative Compounds

**Figure 2:**
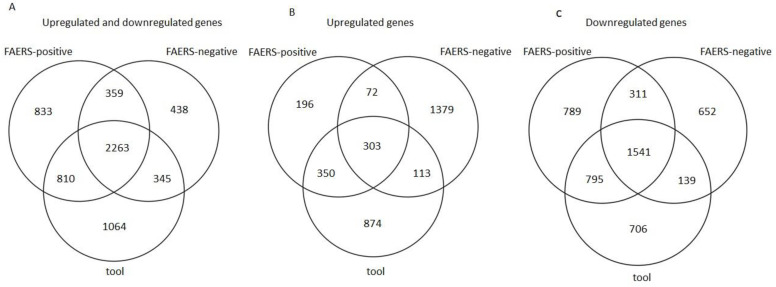
Venn diagrams showing the number of differentially expressed genes (FDR < 0.05 and log2(|FC|) > 1.5) induced by tool, FAERS-positive, and FAERS-negative compounds in rat cortical neuronal cell cultures. (**A**) Both upregulated and downregulated genes. (**B**) Upregulated genes only. (**C**) Downregulated genes only.

Venn diagrams show 2263 common DE genes between the tool, FAERS-positive, and FAERS-negative compounds when both the upregulated and downregulated genes were included in the analysis (Figure 2A). If only the upregulated DE genes were included, 303 DE genes were common to all the compound categories (Figure 2B). If only the downregulated DE genes were included, 1541 DE genes were common to all the compound categories (Figure 2C).

The number of the same DE genes induced by each of the 34 compounds is cross-tabulated in Table 3.

### 2.5. Pathways Enriched by Tool, FAERS-Positive, and FAERS-Negative Compounds

An enrichment analysis of DE genes found a total of 736 enriched Reactome pathways induced by the 34 compounds (Table S1).

The 10 tool compounds induced a total of 333 pathways. Interestingly, all the tool compounds, except pilocarpine, resulted in enriched pathways.

**Figure 3:**
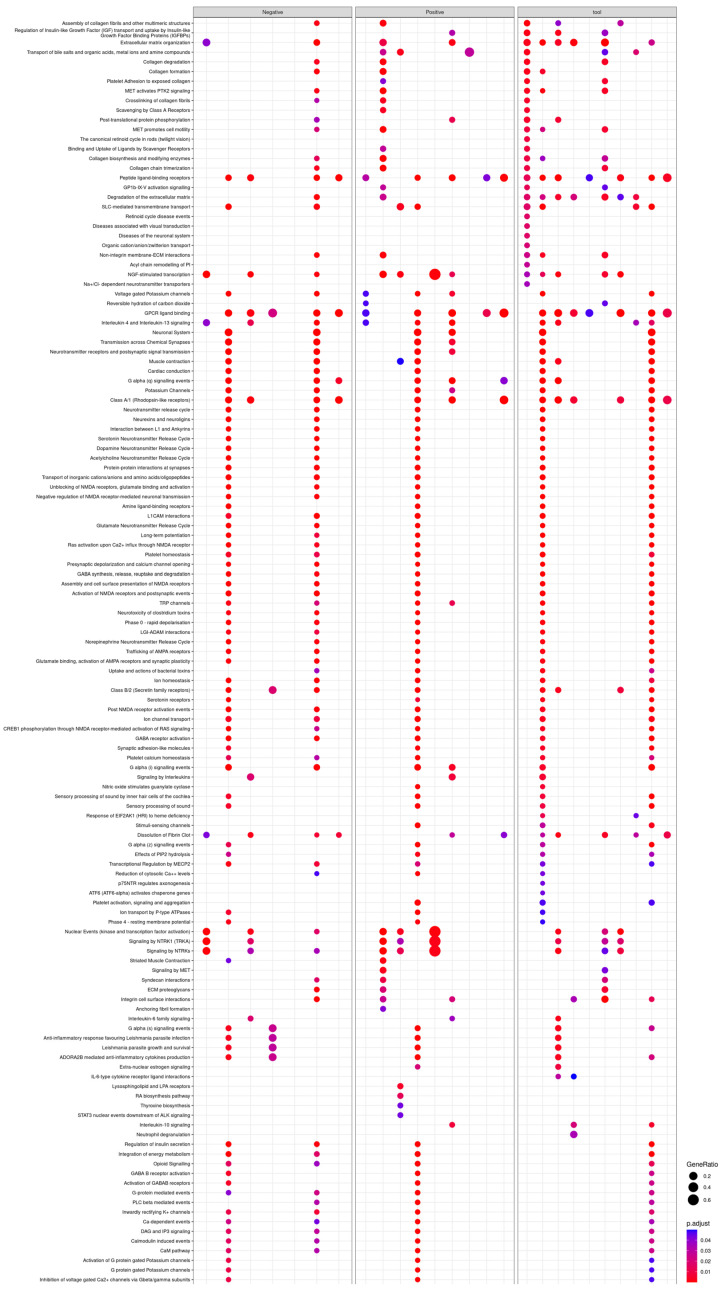

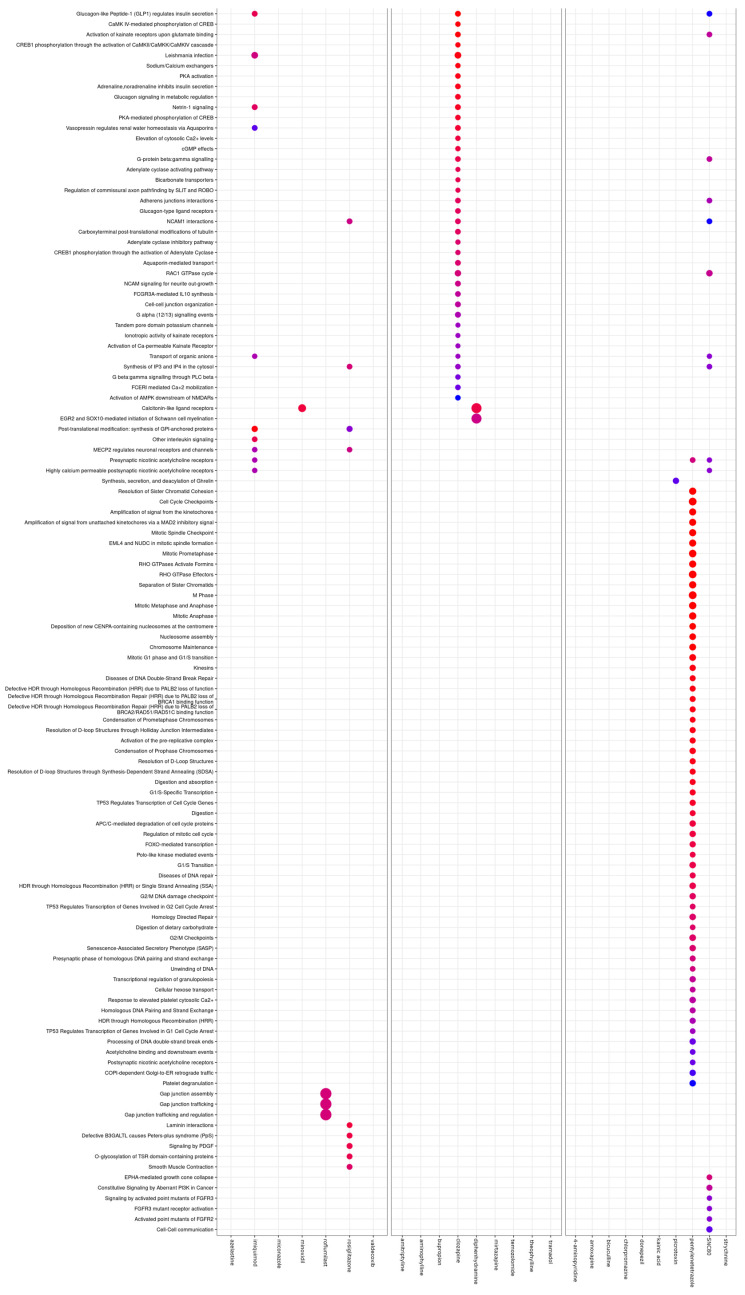
Enriched Reactome pathways (y-axis) induced by tool, FAERS-positive, and FAERS negative compounds (x-axis). Pathways were identified, compared, and visualized by clusterProfiler.

The 13 FAERS-positive compounds induced a total of 197 pathways. Particularly, aminophylline, amitriptyline, bupropion clozapine, diphenhydramine, mirtazapine, temozolimide, theophylline, and tramadol resulted in enriched pathways. Isoniazid, maprotiline, paroxetine, and venlafaxine hydrochloride resulted in no enrichment (Figure 3).

The 11 FAERS-negative compounds induced a total of 206 pathways. The exposure of cultures to azelastine, imiquimod, miconazole, minoxidil, roflumilast, rosiglitazone, or valdecoxib resulted in enriched pathways. Darifenacin, niacin, and ospemifene, however, showed no enrichment.

### 2.6. Gene Ontology of Tool, FAERS-Positive, and FAERS-Negative Compounds

The data are summarized in Table S2.

**Figure 4:**
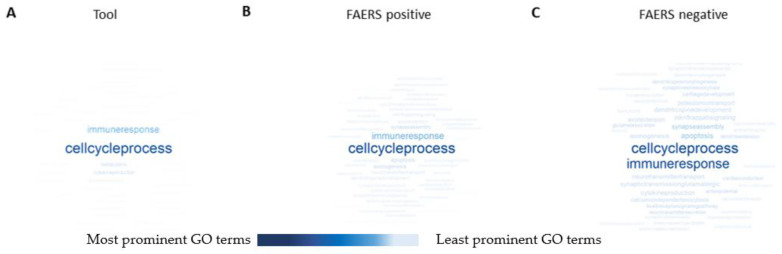
The most prominent gene ontology (GO) terms of the (**A**) tool, (**B**) FAERS-positive, and (**C**) FAERS-negative compounds in a word cloud. The most frequent GO terms have the largest font size and the darkest blue color. In all the compound categories, “cell cycle process” was the most common GO term. In FAERS-positive and FAERS-negative categories, “apoptosis” was also prominent. See Supplementary File S2.

**Tool.** A gene ontology (GO) analysis revealed 4198 enriched GO terms by the tool compounds. Further analysis indicated that 4-aminopyridine had no positively enriched GO terms and picrotoxin and strychnine had no negative GO terms. Word clouds of enriched GO terms revealed “cell cycle process” as the most prominent and “immune response” as the second most prominent term (Figure 4A).

**FAERS-positive**. Altogether, 3232 enriched GO terms were found. Of the FAERS-positive compounds, bupropion had no positively enriched GO terms. Diphenhydramine and temozolomide had no negatively enriched GO terms. Word clouds indicated “cell cycle process” and “immune response” as the most prominent GO terms (Figure 4B).

**FAERS-negative.** Altogether, 3073 enriched GO terms were found. Of the FAERS-negative compounds, darifenacin had no positively enriched GO terms. As with FAERS-positive compounds, word clouds indicated “cell cycle process” and “immune response” as the most prominent GO terms (Figure 4C).

Both the FAERS-positive and FAERS-negative compounds also had enrichment in “apoptosis”, “synapse assembly”, “axonogenesis”, “axon extension”, “synaptic transmission”, and “neurotransmitter transport” GO terms.

### 2.7. Machine-Learning Using Normalized RNA-Sequencing (RNA-Seq) Read Counts Differentiates Tool Compounds from Other Compounds, but Not FAERS-Positive or FAERS-Negative Compounds

**Figure 5:**
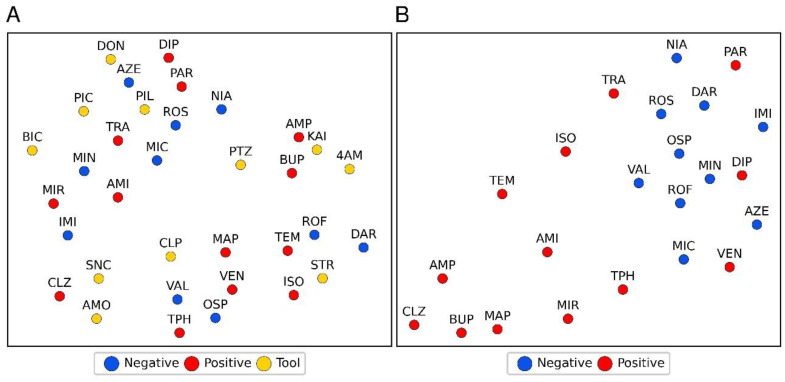
A 2-dimensional illustration of the UMAP reductions of the normalized rank-transformed replicate means of RNA-seq normalized read counts. (**A**) No clustering by compound category (tool, FAERS-positive, FAERS-negative compounds) is apparent. (**B**) A similar reduction for the vectors of a number of differentially expressed genes by FAERS-positive and FAERS-negative with a positive fold-change shared with each tool compound. Abbreviations: The 11 tool compounds: 4-aminopyridine (4AM), amoxapine (AMO), bicuculine (BIC), chlorpromazine (CPL), donepezil (DON), kainic acid (KAI), picrotoxin (PIC), pilocarpine (PIL), pentylenetetrazol (PTZ), strychnine (STR), and SNC80 (SNC). The 13 FAERS-positive compounds: amitriptyline (AMI), aminophylline (AMP), bupropion (BUP), clozapine (CLZ), diphenhydramine (DIP), isoniazid (ISO), maprotiline (MAP), mirtazapine (MIR), paroxetine (PAR), temozolomide (TEM), theophylline (TPH), tramadol (TRA), and venlafaxine (VEN). The 10 FAERS-negative compounds: azelastine (AZE), darifenacin (DAR), imiquimod (IMI), miconazole (MIC), minoxidil (MIN), niacin (NIA), ospemifene (OSP), rosiglitazone (ROS), roflumilast (ROF), and valdecoxib (VAL).

One-vs.-rest machine learning (ML) classification with regularized logistic regression (LR) and support vectors machines (SVM) was applied to assess the separability of compounds in each group (tool, FAERS-positive, FAERS-negative) from the compounds in the remaining groups based on normalized read counts. Table 4 presents the classification performance metrics from the cross-validation. A moderate separation (LR area under the curve (AUC) 0.76 and SVM AUC 0.78) of the tool compounds from the FAERS-positive or FAERS-negative compounds was possible using the normalized RNA-seq counts. The performance in separating FAERS-positive and FAERS-negative compounds from the rest, however, was poor (i.e., FAERS-positive from tool and FAERS-negative; FAERS-negative from tool and FAERS-positive), implying the lack of a clear pattern separating the groups. This lack of clear separation is supported by the UMAP reduction in Figure 5A, where no clear clustering by any of the compound categories was observed.

### 2.8. Machine-Learning Separated FAERS-Positive from FAERS-Negative Compounds Utilizing Differential Gene Expression Shared with Tool Compounds

Next, we assessed the number of tool compounds exhibiting any similarity to the FAERS-positive or FAERS-negative compounds to differentiate the FAERS-positive from the FAERS-negative compounds. Similarity was defined as having one or more DE genes with a positive FC in common with a given tool compound. The leave-one-out cross-validation area under the receiver-operator-characteristic curve for the approach was 0.77 (sensitivity of 0.77, specificity of 1.0, accuracy of 0.87). The *p*-value for accuracy above a no information rate of 0.57 was 0.002. The approach correctly classified 10 of the 13 FAERS-positive compounds. The exceptions included paroxetine, diphenhydramine, and venlafaxine, which were incorrectly classified as FAERS-negative compounds. Figure 5B shows the UMAP reduction for vectors, consisting of the number of DE genes with a positive FC shared by each FAERS-positive and-negative compound with different tool compounds. The separation between the FAERS-positive and FAERS-negative compounds is significantly clearer in this features space than in the space of normalized counts (Figure 5A).

### 2.9. Alikeness-% and Gene Set Enrichment Analysis (GSEA) Score

**Alikeness-%.** The alikeness-% was generated to compare the number of common DE genes between the FAERS-positive and tool compounds. The alikeness-% sum for clozapine was 221.5, which was the highest sum among all 13 FAERS-positive compounds (Table 5). Mirtazapine, aminophylline, and bupropion had comparable alikeness-% sums, ranging from 50 to 70. Maprotiline, theophylline, amitriptyline, temozolomide, tramadol, and venlafaxine hydrochloride had alikeness-% sums ranging from 5 to 50. As expected by the low number of DE genes, isoniazid, paroxetine, and diphenhydramine had alikeness-% sums <1, indicating that, similar to tool compounds, they did not induce gene expression changes.

**GSEA score.** Clozapine had a GSEA score of 28.8, which was the highest of all the FAERS-positive compounds (Table 6). Mirtazapine had the next highest score, 20.1. Bupropion and amitriptyline had a GSEA score within a comparable range, from 7 to 12. The theophylline, maprotiline, and venlafaxine hydrochloride scores ranged from 5 to 3. Interestingly, clozapine, mirtazapine, and bupropion were the top three FAERS-positive compounds with a high alikeness-% sum (Table 5). No GSEA score could be calculated for tramadol, temozolomide, isoniazid, aminophylline, or diphenhydramine.

**Comparison of the alikeness-% sum and the GSEA score**. The Spearman correlation of the alikeness-% sum and GSEA score was 0.889 (*p* = 4.911 × 10^−5^).

**Taken together.** Alikeness-% correctly classified 9 of the 13 FAERS-positive compounds when the cut-off for the alikeness-% sum was set to >10%. The GSEA score correctly classified 8 of the 13 FAERS-positive compounds (Table 7). Amitriptyline, aminophylline, bupropion, clozapine, maprotiline, mirtazapine, and theophylline (7 of the 13 compounds) were classified correctly by both scoring systems. Alikeness-% also correctly classified temozolomide and tramadol, which were not detected by the GSEA score. The GSEA score detected venlafaxine hydrochloride, which had an alikeness-% of 7.4%. Both scoring systems failed to detect diphenhydramine (three DE genes) and paroxetine (no DE genes). Machine learning failed to correctly classify diphenhydramine and paroxetine as well as venlafaxine hydrochloride (151 DE genes). Importantly, the falsely classified compounds had a very small number or no DE genes (Table 2). Isoniazid with only 32 DE genes, however, was correctly classified into the FAERS-positive category by the machine-learning approach but not by the alikeness-% or the GSEA score. If the two compounds (diphenhydramine with three DE genes, paroxetine with no DE genes) were excluded from the FAERS-positive category originally containing 13 compounds, the alikeness-% correctly classified 9 of 11 (isoniazid and venlafaxine incorrectly categorized), the GSEA score correctly classified 8 of 11 (isoniazid, temozolomide, and tramadol incorrectly categorized) and the machine-learning approach correctly classified 10 of 11 (venlafaxine incorrectly categorized) of the FAERS-positive compounds.

## 3. Discussion

The present study aimed to develop a transcriptomics-based in vitro assay for the early detection of drug-induced seizure liability. Rat cortical neuronal cultures were exposed for 24 h to non-toxic concentrations of compounds with known, suspected, or no seizure liability. The RNA-seq data were analyzed using bioinformatics and machine-learning approaches. We had four major findings. We hypothesized that the gene expression profile of the FAERS-positive compounds would be comparable to that of the tool compounds. (1) Each of the tool compounds with a known ictogenic effect induced a compound-specific transcriptomics signature. We were unable to identify any “common ictogenic signature”. (2) Also, the FAERS-negative compounds with no or very low seizure liability induced a remarkable gene expression profile, which was considered “noise” in the analysis platform. (3) The one-by-one comparison of DE genes between the FAERS-positive and tool compounds revealed up to 61.9% similarity (clozapine vs. SNC80). Clozapine had the highest alikeness-% sum, showing >5% alikeness-% with 8 of the 11 tool compounds. (4) Unexpectedly, 2 of the 13 FAERS-positive compounds (diphenhydramine and paroxetine) induced no significant DE genes. Consequently, the alikeness-% correctly categorized 82%, the GSEA score correctly categorized 73%, and the machine-learning approach correctly categorized 91% of the remaining 11 FAERS-positive compounds.

### 3.1. Gene Expression in Rat Cortical Cell Cultures by Tool, FAERS-Positive, and FAERS-Negative Compounds Was Not Associated with Neurotoxicity

The FAERS-positive and FAERS-negative compounds and even some of the tool compounds (pilocarpine, pentylenetetrazol, 4-aminopyridine, chlorpromazine, amoxapine, and donezepil) are administered clinically to treat various diseases in the brain or peripheral tissues or for diagnostic purposes. Consequently, defining the optimal concentration for in vivo gene expression studies in neuronal cell cultures of this heterogeneous list of compounds was challenging [30,31]. First, the dose selection was guided by the information available on the therapeutic plasma concentrations. Second, we tested the neurotoxicity of each compound around the therapeutic concentration range and selected the highest tolerated concentration in our assay, as our preliminary studies with 4-aminopyridine, bupropion, kainic acid, and pentylenetetrazol indicated that the drug-induced gene expression was highly concentration-dependent.

Of the 34 compounds tested, only maprotiline (30 µM), miconazole (30 and 100 µM), chlorpromazine (100 µM), and kainic acid (100 µM) induced neurotoxicity in rat cortical neuronal cell cultures, as indicated by the increased lactate dehydrogenase (LDH) levels in the conditioned medium. Consistent with the present data, kainic acid was reported to reduce the cell viability in mouse cortical neuronal cultures at concentrations between 10 and 1000 µM [32]. Furthermore, maprotiline reduced the viability of neuro-2a cell cultures at concentrations between 5 and 100 µM [33]. Based on the toxicity information, the drug concentrations were adjusted to the highest non-toxic levels to minimize the lack of induced expression, which was needed for the design of the assay.

### 3.2. The Tool, FAERS-Positive, and FAERS-Negative Compounds Induce Category-Specific Gene Expression, but Pathway and GO Analyses Revealed No Specific Markers for Seizure Liability

First, we compared the gene expression generated by all the compounds included in the tool, FAERS-positive, or FAERS-negative categories. Our aim was to identify an “ictogenic gene expression signature” that would be induced by all or a majority of the tool compounds, and then to use the signature to categorize the FAERS-positive compounds as “potentially” ictogenic and the FAERS-negative compounds as “non-ictogenic” based on their gene expression profile.

Venn diagrams indicated that the three compound categories had an overlap of over 2000 DE genes. The tool compounds, however, also had DE genes specific to that compound category, suggesting that gene expression might indeed be able to differentiate the risk of ictogenicity.

Next, we investigated whether the gene expression profiles of the FAERS-positive and FAERS negative compounds had pathways or GO terms comparable to those of the tool compounds, which would reveal the presence or lack of their seizure-inducing properties.

Although the pathway analysis identified several epilepsy- and seizure-related pathways, it did not identify any pathways specific to the tool, FAERS-positive, or FAERS-negative compounds. For example, some tool (amoxapine, SNC80), FAERS-positive (clozapine), and FAERS-negative (imiquimod) compounds induced pathways related to GABA, an inhibitory amino acid regulating neuronal excitability. Some tool (amoxapine, SNC80), FAERS-positive (clozapine), as well as FAERS-negative (imiquimod, rosiglitazone) compounds induced response element binding protein (CREB)-related pathways involved in regulating gene expression changes in epilepsy [34]. In addition, some tool (amoxapine, SNC80), FAERS-positive (amitriptyline, clozapine, mirtazapine), and FAERS-negative (imiquimod, rosiglitazone) compounds induced voltage-gated ion channel-related pathways, contributing to ictogenesis in both in vitro and in vivo models [35,36]. Finally, some tool (amoxapine, SNC80), FAERS-positive (clozapine), and FAERS-negative (imiquimod, rosiglitazone) compounds induced α-amino-3-hydroxy-5-methyl-4-isoxazolepropionic acid receptor (AMPA)-related pathways [37].

Contrary to our expectations, the analysis of GO terms in the tool compound category revealed no seizure-related terms. For example, 4-aminopyridine, amoxapine, bicuculline, chlorpromazine, donepezil, kainic acid, picrotoxin, and SNC80 indicated “aorta morphogenesis” and “collagen metabolic process” terms. The FAERS-positive drugs, however, indicated several GO terms that could indicate the risk of seizures, e.g., aminophylline, amitriptyline, clozapine, maprotiline, mirtazapine, theophylline, tramadol, and venlafaxine were linked to the terms “calcium ion regulated exocytosis of neurotransmitter” and “regulation of membrane potential”. Furthermore, the FAERS-negative compounds azelastine, imiquimod, miconazole, minoxidil, ospemifene, rosiglitazone, and valdecoxib were linked to the terms “regulation of neurotransmitter levels” and “regulation of postsynaptic membrane potential”, which could indicate the molecular pathways related to seizure liability. In general, the enriched GO terms indicated that the most common GO terms in the tool, FAERS-positive, and FAERS-negative categories were “cell cycle process” and “immune response”. Interestingly, both of these processes relate to the acute phase of status epilepticus, epileptogenesis, and epilepsy [38,39,40].

Taken together, even though the gene expression profiles of the tool, FAERS-positive, and FAERS-negative compounds were linked to several enriched pathways and GO terms potentially signaling for seizure liability, they were not specific to any compound category.

### 3.3. From a Common to Drug-Specific Ictogenic Signature

As the pathway analysis indicated compound-specific rather than compound-common gene expression profiles, we next compared the gene expression of each FAERS-positive compound to the 11 tool compounds one-by-one using three different strategies.

Alikeness-% indicated the percentage of similar DE genes between the two compounds. The sum of the alikeness-% informed us of the similarity to all 11 tool compounds, and was the highest for clozapine, which showed overlapping gene expression with 9 of the 11 tool compounds. Importantly, the basic assumption was that the compound had to induce DE gene expression. Contrary to our expectations, 2 of the 13 FAERS-positive compounds induced no (paroxetine) or a very low number (three, diphenhydramine) of DE genes. After their exclusion, 9 of the 11 remaining FAERS-positive drugs had a sum alikeness-% ≥ 10%, indicating a DE gene expression similar to that of one or more tool compounds with known seizure liability. The 32 DE genes induced by isoniazid with the lowest sum alikeness-% of 0.3 had almost no similarity to any of the tool compounds, suggesting different/novel mechanisms for its seizure liability. The 151 DE genes induced by venlafaxine had a sum alikeness-% of 7.4, of which the highest single alikeness-% (2.4) was with SNC80.

The GSEA score takes into account the similarity in both the upregulated and downregulated genes between the FAERS-positive and tool compounds. The analysis revealed that 8 of the 13 FAERS-positive compounds showed similar gene regulation to one or more tool compounds. After the exclusion of the two low-inducing FAERS-positive compounds, the GSEA analysis found similarities between 8 of the 11 FAERS-positive compounds and the tool compounds. Isoniazid (32 DE genes), temozolomide (21 DE genes), and tramadol (100 DE genes) were undetected by the GSEA analysis.

The machine-learning approach correctly classified 10 of the 13 FAERS-positive compounds from the FAERS-negative compounds. After the exclusion of the two low-inducing FAERS-positive compounds, machine learning correctly separated 10 of the 11 FAERS positive compounds from the FAERS-negative compounds. The approach did not differentiate venlafaxine as a FAERS-positive compound.

Finally, the rankings of the compounds by the different approaches were comparable. For example, both the alikeness-% and the GSEA score were highest for clozapine and mirtazapine. Venlafaxine was not found by the alikeness-% or machine-learning approaches and was ranked lowest by the GSEA score.

## 4. Materials and Methods

### 4.1. Rat Primary Cortical Cell Cultures

**Figure 6:**
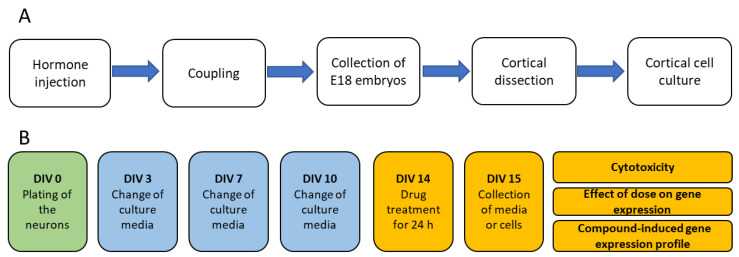
Production of embryos and set-up of cell cultures. (**A**) Production of embryos for the rat primary cortical cell cultures. (**B**) Treatment of cell cultures for the assessment of compound-induced cytotoxicity, dose-optimization, and compound-induced gene expression.

Primary cortical cell cultures were used for the cytotoxicity assay, compound dose optimization assay, and the assessment of compound-induced gene expression. The cell culturing pipeline is summarized in Figure 6.

#### 4.1.1. Production of Embryos

Adult female Sprague-Dawley rats of breeding age (Envigo, Horst, Limburg, The Netherlands) were injected with the luteinizing hormone-releasing hormone (LHRH) agonist (#L4513, Sigma-Aldrich, St. Louis, MO, USA; single injection of 40 µg, i.p.) to ensure an optimal menstrual cycle for coupling. Five days after the LHRH injection, the females were coupled overnight with the male rats (Figure 6), which was counted as embryotic day 0 (E0). On E18, the rats were anesthetized by the inhalation of 5% isoflurane, the neck was dislocated, and the embryos were collected to dissect the cerebral cortex for the cell cultures.

For the cytotoxicity experiment, four cell culture batches were generated. In batch #1, three females produced 38 embryos; in batch #2, three females produced 29 embryos; in batch #3, four females produced 52 embryos; and in batch #4, four females produced 49 embryos. The cerebral cortex was dissected and plated on 96-well plates.

For the compound dose optimization experiment, three female rats produced 45 embryos. The cortical cells were plated on 24-well plates.

For the compound-induced gene expression experiment, four female rats produced 49 embryos. The cortical cells were plated on 24-well plates.

#### 4.1.2. Dissection of the Cerebral Cortex and Cell Plating

The cerebral cortex of each of the E18 embryos was dissected and the neuronal cells were plated as described earlier [41,42]. Briefly, the cortex was dissected under a stereomicroscope in Dulbecco’s modified Eagles medium (DMEM; #BE12-614F, Lonza, Basel, Switzerland), supplemented with 10% fetal bovine serum (#10270-106, Thermo Fisher Scientific, Waltham, MA, USA) and 2 mM l-glutamine (#BE17-605E, Lonza). The cortices were rinsed with HC-buffer (1× phosphate-buffered saline (#70013032, Gibco, Grand Island, NY, USA) containing 1 mg/mL bovine serum albumin and 10 mM glucose), and then, trypsin-digested using 0.125% trypsin-DMEM for 20 min at 37 °C. Trypsin (#15090-046, Thermo Fisher Scientific) digestion was stopped with an equivalent volume of DMEM, containing 10% fetal bovine serum, 100 U/mL penicillin, and 100 μg/mL streptomycin (#DE17-602E, Lonza). To obtain a single-cell suspension, the tissue was centrifuged (1600 g, 5 min, room temperature), suspended in the plating medium with a pipette, and incubated at room temperature for 2 min. The cell suspension was filtered through a 40-μm filter (#542 040, Greiner Bio-One, Kremsmünster, Austria) to remove the remaining tissue, and then centrifuged (1200× *g*, 5 min, room temperature) to remove the plating medium. The neurons in the cell suspension were counted with a hemocytometer (#DHC-01, NanoEnTek, Seoul, Korea) and plated on poly-d-lysine-coated 24 or 96-well plates (#P6407, Sigma-Aldrich, St. Louis, MO, USA) in a neurobasal-medium supplemented with B27 (2%, #17504-044, Thermo Fisher Scientific), 100 U/mL penicillin, 100 μg/mL streptomycin (#DE17-602E, Lonza), and 2 mM L-glutamine (1%, (#17-605E, Lonza) at a density of 3 × 10^5^ cells/mL (i.e., 1.6 × 10^5^ cells/cm^2^).

### 4.2. Compound Selection

The 11 tool compounds were selected based on their known ictogenic properties and different mechanisms of action. The 13 FAERS-positive test compounds included those frequently associated with seizures based on the FAERS database and the VigiAccess adverse event records [29] followed by the disproportionality analysis [43]. The 10 FAERS-negative compounds exhibited very low or no linkage with seizures in the analysis.

### 4.3. Assessment of Cytotoxicity

To determine the highest tolerated (non-toxic) concentration of the 11 tool, 13 FAERS-positive, and 10 FAERSnegative compounds, the cortical cell cultures were incubated with each drug for 24 h and the amount of LDH was measured as an indicator of cytotoxicity. Four drug concentrations and four replicate wells were analyzed. The range of drug concentrations included the known therapeutic plasma concentration in humans, which was determined by a literature review (Table 8).

The LDH assay was performed using the Cytotoxicity Detection Kit (#11644793001, Roche, Basel, Switzerland) as described previously [44]. Briefly, the culture medium was collected from the 96-well cell culture plate, pipetted to a 96-well microplate, and then centrifuged (300 g, 3 min, room temperature) to pellet the possible cell remains to the bottom. The supernatant was then diluted 1:4 with distilled water and the reaction mixture was prepared by mixing a dye solution and a catalyst at a ratio of 1:45. Then, 100 µl of the diluted supernatant and 100 µL of the reaction mixture were pipetted onto the 96-well plate and incubated in the dark for 30 min at room temperature. Finally, the absorbance was measured at 490 nm with a microplate reader (Wallac Victor^2^, Perkin Elmer, CA, USA).

The four wells incubated without any treatment or only with vehicle (DMSO 0.1%) served as a “low control”. The four wells treated with Triton X-100 (#93418, Sigma), leading to a maximal LDH release, served as a “high control”. To calculate the magnitude of cytotoxicity % in each treated well, the following formula was used:

(1) $$Cytotoxicity\;\%=\frac{absorbance\;of\;treated\;well-average\;absorbace\;of\;low\;control\;wells}{average\;absorbance\;of\;high\;control\;wells-average\;absorbance\;of\;low\;control\;wells}\times 100$$

The statistical significance of the difference between the compound- and vehicle-induced cytotoxicity was assessed with the Kruskal–Wallis test, followed by a post hoc analysis using the Mann–Whitney U test in GraphPad Prism software (version 9.3.1).

### 4.4. Effect of Compound Dose on Gene Expression and the Compound-Induced Gene Expression Profile

Next, we determined the gene expression profile of each of the 34 compounds in the rat cortical neuronal cell cultures. After a 24-h drug exposure, the cells were collected from the 24-well plates, the total RNA was extracted, the RNA-sequencing library was constructed, and the RNA was sequenced using the Illumina platform (see Section 4.4.3 for the sequencing library construction and sequencing details).

#### 4.4.1. Extraction of Total RNA

The RNA was extracted with an RNeasy mini kit (#74104; Qiagen, Hilden, Germany) according to the manufacturer’s instructions. Briefly, after a 24-h incubation with the drugs, the cell culture media were removed from the wells (four replicates) and replaced with 350 µL of RLT buffer containing 1% of mercaptoethanol (#M6250, Sigma). The cells were scraped into the RLT buffer with a cell scraper (#83.1832, Sarstedt, Newton, NC, USA) and transferred to a 1.5-Ml centrifuge tube. The cells were homogenized by vortexing for 1 min. Then, 350 µL of 70% ethanol was added to the lysate. The lysate was then transferred to a column, centrifuged, and washed with RW1 buffer. For the DNA removal, DNAse I (#79256, Qiagen) and an RDD buffer mixture were added to the column and the mixture was incubated at room temperature for 15 min. The column was washed with RW1 and an RPE buffer according to the manufacturer’s protocol. The remaining RNA was eluted to 50 µL of RNase-free water, aliquoted, and stored at −70 °C for quality control and RNA-seq.

#### 4.4.2. Quality Control of Extracted RNA

The RNA concentration and RNA integrity number (RIN) were measured with a 2100 Bioanalyzer (Agilent, Santa Clara, CA, USA) using the RNA 6000 Nano Kit (#5067–1511, Agilent).

In the compound dose optimization experiment, the median concentration of the extracted RNA was 102.8 ng/µL (range 39.4–153.3 ng/µL) and the median RIN was 8.8 (range 7.7–9.6).

In the compound-induced gene expression experiment, the median concentration of the extracted RNA was 37.4 ng/µL (range 15.8–153.4) and the median RIN was 9.3 (range 7.7–10).

#### 4.4.3. mRNA-seq Library and Sequencing

In the compound dose optimization experiment, 300 ng of the total RNA was used to prepare the sequencing library using a TruSeq Stranded mRNA HT Kit (#15031047, Illumina, San Diego, CA, USA). The sequencing was conducted using a NovaSeq 6000 instrument (Illumina) with 2 × 50 bp paired end reads targeting 15–18 M raw reads per replicate.

In the compound-induced gene expression experiment, 100 ng of the total RNA was used to prepare the sequencing library using an Illumina Stranded mRNA Preparation kit (#1000000124518, Illumina).

The sequencing was performed using the NovaSeq 6000 instrument (Illumina) with 2 × 100 bp paired end reads, aiming at 27–33 M raw reads per replicate.

### 4.5. Bioinformatics

#### 4.5.1. Quality Control and Mapping of mRNA Sequencing Data

The details of the mRNA-seq data quality control, mapping, and identification of the DE genes have been previously described [45]. Briefly, the quality control of the mRNA-seq reads was performed using FastQC and MultiQC to define the quality score and the number of produced reads [46,47]. The reads were mapped to the Ensemble RN6 genome (Rnor 6.0.99) with STAR software (version 2.7.3a) [48]. In the dose-effect experiment, the median of the mapped reads was 17.4 M (range 14.2–21.1 M). In the gene expression experiment, the median of the mapped reads was 34.9 M (range 8.7–69.7 M).

#### 4.5.2. Identification of DE Genes

The DE genes were identified using the DEseq2 [49] R package (R version 4.1.0). The Benjamini–Hochberg false discovery rate (FDR) was used to calculate the adjusted *p*-value. The differences in the gene expression between the drug-exposed and vehicle-exposed cultures were considered significant if the FDR was <0.05 and the log2(|FC|) was >1.5. Normalized read counts in fragments per kilobase of per million mapped reads (FPKM) were extracted with the fpkm-function in DeSeq2 for further use in machine-learning analysis.

#### 4.5.3. Comparison of the Number of DE Genes between the Tool, FAERS-Positive, and FAERS-Negative Compounds

Venn diagrams were generated using the VennDiagram R package in R to assess the similarity in the gene expression profiles induced by the tool, FAERS-positive, and FAERS-negative compounds [50]. A gene was classified into the tool, FAERS-negative, or FAERS-positive category if its differential expression was induced by at least one compound in a given compound category.

A cross-tabulation table, showing the number of DE genes between all the 34 tested compounds was generated to assess the similarity of the drug-induced gene expression between the compound categories (Table 3).

#### 4.5.4. Identification of Enriched Pathways

The ClusterProfiler R-package was used to identify enriched pathways, compare identified pathways between the tool, FAERS-positive, and FAERS-negative compounds, and to visualize the results [51]. The pathway identification was performed using pathways in Reactome in the ClusterProfiler [52]. A pathway was considered enriched if the FDR was <0.05.

#### 4.5.5. Gene Ontology Analysis of DE Genes

To identify the enriched GO terms of the DE genes for each compound, the GSEA was performed using the Molecular Signatures Database (MSigDB). The rat IDs of the DE genes were converted to their human homologues in the Ensembl Biomart web interface [53]. To generate the rank list, the DE genes were ranked according to the log2(|FC|), which were then loaded into the GSEA software [54]. In the GSEA, enriched GO terms were obtained for each ranked list by performing a GSEA pre-ranked analysis using the biologic processes gene sets (BP-subset of C5 category, version 7.5) with 1000 permutations, and no collapse option [55].

To identify the enriched GO terms (FDR < 0.05) for each tool, FAERS-positive and FAERS-negative compound, the enriched terms were analyzed with Cytoscape (version 3.9, FDR < 0.05) [56]. Then, to visualize the GO terms related to each compound category, word clouds were generated in R using the word clouds package [57,58]. To simplify the wording in the word clouds, the GO terms were abbreviated. That is, the prefix GOBP was removed, and then interleukin (IL), G protein-coupled receptor (GPCR), transforming growth factor (TGF), tumor necrosis factor (TNF), extracellular matrix (ECM), reactive oxygen species (ROS), and endoplasmic reticulum (ER) were replaced with abbreviations. The GO terms “apoptotic process” or “cell-mediated apoptotic process” were shortened to “apoptosis”. “Regulation of and positive/negative regulation” was removed from all the GO terms. The GO terms with “cell-type–mediated cytokine production” were shortened to “cytokine production” and the GO terms with “cell-type–mediated immune response” were shortened to “immune response”; “metabolic process” was shortened to “metabolism” and “different cell cycle phases” was shortened to “cell cycle process”.

#### 4.5.6. Alikeness-% and GSEA Score

The gene expression “alikeness-%” was generated to determine the likelihood of a given compound having ictogenic properties based on the DE genes. To reduce noise in the alikeness-% (i.e., changes in the gene expression profile that do not contribute to ictogenicity), the DE genes of the FAERS-negative compounds, which were also regulated by the tool or FAERS-positive compounds, were removed (Figure 2). Next, the DE genes with the same direction of regulation by both the drug of interest and the tool compound (e.g., upregulated by both a given FAERS-positive compound and any tool compound) were identified. Then, the alikeness-% for a given FAERS-positive compound with any of the tool compounds was calculated as follows: (number of the same DE genes with a tool compound/number of all DE genes induced by the tool compound) × 100%. This was repeated for all the combinations of FAERS-positive and tool compounds (Table 5).

As another approach to identify ictogenic properties in the gene expression profile, we generated the GSEA score. As for the generation of the alikeness-%, the DE genes of the FAERS-negative compounds only were removed from the expression profiles of the FAERS-positive and tool compounds. Then, the GSEA rank lists were generated from the gene expression data available from the tool compounds by ranking the genes in order according to the *p*-value of the mRNA differential expression. The upregulated genes were assigned a positive rank number and the downregulated genes a negative rank number. Then, two gene sets were generated for all the FAERS-positive compounds: one for the upregulated DE genes and another for the downregulated DE genes. Finally, to assess enrichment, all the gene sets were compared to all the ranked lists with the fgsea R-package, and significant normalized enrichment scores (adjusted *p*-value < 0.05) were included with the GSEA scores (Table 6) [59].

#### 4.5.7. Machine-Learning Analysis

##### Classification Using Normalized RNA-Seq Counts

Prior to classification, the low-expressing genes were filtered by removing genes with fewer than five normalized counts (FPKM) in at least 50% of the samples. This reduced the number of genes from 32,623 to 10,326. The classification was performed using a pipeline, including three stages: (1) feature pre-processing, (2) feature selection, and (3) classification. In the feature pre-processing step, the counts from each sample were rank-transformed [60] and the per-gene median of the ranks over the four replicates was calculated for each compound. The medians were standardized to a zero mean and unit variance over the dataset. In the feature selection step, the genes were filtered by selecting the k genes with the highest F-value (the ratio of the variance of group means over the mean of within-group variances). The value for k and whether to omit the filtering step were selected during the pipeline hyperparameter optimization. In the classification step, the compound was classified using logistic regression (LR) with l1 or l2 regularization [61] and support vector machines (SVM) [62] with linear or radial basis kernels. The mode and strength of regularization for the LR and SVM kernel along with the regularization strength were selected during the hyperparameter optimization.

The classification performance was evaluated using nested stratified cross-validation, where all the compounds (i.e., tool, FAERS-positive, and FAERS-negative compounds) were repeatedly split into testing and training sets. The testing set contained a single compound from one of the three compound categories (target group) and three to four randomly sampled compounds from the entire (non-target) compound set. The training set contained the remaining compounds that were not included in the testing set. In the outer cross-validation loop, each testing set was classified utilizing a classifier trained on the compounds in the training set. The predicted classes and actual classes were pooled over the outer cross-validation to calculate the classification performance metrics. The pipeline hyperparameters were optimized in an inner leave-one-out cross-validation loop performed over the training compounds. The classification pipeline and cross-validation were implemented using Python 3.10.6, Sklearn 1.0.2, and Numpy 1.2.

##### Classification Utilizing DE Genes Shared with Tool Compounds

The set of DE genes with a positive FC > 1.5 was determined for each compound using DeSeq2 and FC shrinkage via apeglm [63]. All the DE genes expressed by the FAERS-negative compounds were subtracted from the DE gene list. Then, the number of tool compounds sharing at least one DE gene with the FAERS-positive or FAERS-negative compounds was calculated. Next, the predictive power was evaluated using leave-one-out cross-validation, where each compound in the combined set of FAERS-positive or -negative compounds was left out one-by-one. A logistic regression model with the constant was trained on the number of tool compounds with at least one similar DE gene. The DE analysis and classification were performed using R 4.1.3.

##### Data Visualization

A dimensionality reduction using UMAP to 2-dimensional space, preceded by a standardization to zero mean and unit variance per feature, was performed for the visualization of the high-dimensional data. For the normalized RNA-seq counts, PCA to 30D space was performed prior to reduction.

## 5. Conclusions

Alikeness-% correctly categorized 85%, the GSEA score correctly categorized 73%, and the machine-learning approach correctly categorized 91% of the FAERS-positive compounds with reported seizure liability in clinical use. Several factors can explain the incorrect prediction of seizure liability. These include a low number of induced DE genes, the heterogeneity of the mechanism of action of the compounds, and the heterogeneity of the induced gene expression by different compounds. Only one exposure duration was used, which may not completely capture the full expression profile of the different compounds. In addition, the number of tool compounds was rather small, representing a limited spectrum of ictogenic mechanisms and gene expression patterns for comparison. Furthermore, some seizure liability mechanisms and gene expression patterns remain to be discovered. Finally, the set of test drugs (FAERS-positive compounds) with reported seizure liability were chosen from the FAERS adverse event database [28], leaving some uncertainty regarding whether the ictogenic properties of the compound were related to the condition treated by the drug rather than the drug itself, and consequently, would present a false positive in the FAERS compound category. A larger number of tool compounds with known seizure liability could further improve the sensitivity and specificity of the test platform. Moreover, the expansion of the list of FAERS-negative compounds would help to remove the non-ictogenicity -related “noise” in the DE gene list of tool and test compounds. Overall, gene expression-based analysis shows promise as a novel tool to predict the seizure liability of novel compounds.

## Figures and Tables

**Table 1:** Number of differentially expressed genes.

Compound	Concentration (µM)	Number of DE Genes
4-aminopyridine	1	0
	100	88
aminophylline	1	0
	100	0
bupropion	1	0
	100	25
kainic acid	0.1	0
	10	7
pentylenetetrazol	1000	0
	20,000	851

Number of differentially expressed genes (FDR < 0.05 and log2(|FC|) > 1.5) in rat cortical cell culture after 24-h of incubation with two concentrations of 4-aminopyridine, aminophylline, bupropion, kainic acid, or pentylenetetrazol. Abbreviations: DE, differentially expressed; FC, fold-change; FDR, false discovery rate.

**Table 2:** Number of differentially expressed genes.

Drug	Concentration (µM)	Number of DE Genes	Group
4-aminopyridine	100	340	tool
amoxapine	30	2659	tool
bicuculline	100	657	tool
chlorpromazine	10	305	tool
donepezil	10	141	tool
kainic acid	10	162	tool
picrotoxin	30	273	tool
pilocarpine HCl	10	21	tool
pentylenetetrazol	20,000	1014	tool
SNC80	30	2308	tool
strychnine	10	60	tool
amitriptyline	10	346	FAERS-positive
aminophylline	100	158	FAERS-positive
bupropion	100	202	FAERS-positive
clozapine	30	3695	FAERS-positive
diphenhydramine	10	3	FAERS-positive
isoniazid	100	32	FAERS-positive
maprotiline	10	141	FAERS-positive
mirtazapine	30	827	FAERS-positive
paroxetine	10	0	FAERS-positive
temozolomide	30	21	FAERS-positive
theophylline	100	200	FAERS-positive
tramadol	30	100	FAERS-positive
venlafaxine hydrochloride	10	151	FAERS-positive
azelastine	10	84	FAERS-negative
darifenacin	1	23	FAERS-negative
imiquimod	1	2214	FAERS-negative
miconazole	10	332	FAERS-negative
minoxidil	30	36	FAERS-negative
niacin	30	15	FAERS-negative
ospemifene	30	259	FAERS-negative
roflumilast	10	7	FAERS-negative
rosiglitazone *	100	1827	FAERS-negative
valdecoxib	30	479	FAERS-negative
DMSO 0.1%	1%	1686	Vehicle

Number of differentially expressed genes (FDR < 0.05 and log2(|FC|) > 1.5) in rat cortical cell culture after 24-h of incubation with tool, FAERS-positive, and FAERS-negative compounds. Vehicle refers to the incubation of cells in DMSO 0.1% or 1%. * 1% DMSO (dimethylsulfoxide) was used as a vehicle. Other compounds were dissolved in 0.1% DMSO. Abbreviations: DE, differentially expressed; FC, fold-change; FDR, false discovery rate.

**Table 3:** Cross-tabulation of the differentially expressed (DE) genes (FDR < 0.05 and log2(|FC|) > 1.5) induced by the tool, FAERS-positive, and FAERS-negative compounds.

	4-Aminopyridine	Amoxapine	Bicuculline	Chlorpromazine	Donepezil	Kainic Acid	Picrotoxin	Pilocarpine HCl	Pentylenetetrazole	SNC80	Strychnine	Amitriptyline	Aminophyline	Bupropion	Clozapine	Diphenhydramine	Isoniazid	Maprotiline	Mirtazapine	Paroxetine	Temozolomide	Theophylline	Tramadol HCl	Venlafaxine HCl	Azelastine	Darifenacin	Imiquimod	Miconazole	Minoxidil	Niacin	Ospemifene	Roflumilast	Rosiglitazone	Valdecoxib	DMSO
4-aminopyridine	340	109	60	30	14	70	41	1	95	77	10	41	72	50	128	1	4	22	45	0	6	22	19	9	10	5	92	34	4	0	21	1	81	21	79
amoxapine	109	2659	388	202	86	56	137	15	278	1560	32	172	54	71	2006	1	15	116	555	0	11	90	48	71	48	12	1190	232	19	12	135	3	1274	315	1173
bicuculline	60	388	657	93	67	33	173	10	133	372	38	98	38	47	475	2	7	53	274	0	3	64	43	39	44	15	297	130	16	8	93	2	371	153	387
chlorpromazine	30	202	93	305	53	24	28	16	70	200	24	96	18	34	200	2	17	92	148	0	8	52	20	33	26	11	114	113	10	14	77	4	147	133	149
donepezil	14	86	67	53	141	13	20	7	41	91	8	42	12	8	88	2	4	32	78	0	4	26	20	10	25	14	57	66	7	7	25	2	77	51	78
kainic acid	70	56	33	24	13	162	17	2	50	45	8	26	59	40	56	1	2	15	36	0	4	14	10	5	14	7	46	30	7	2	22	1	39	22	40
picrotoxin	41	137	173	28	20	17	273	1	60	132	30	46	23	35	169	2	7	18	93	0	3	46	32	34	23	10	118	43	13	3	48	2	120	51	137
pilocarpine HCl	1	15	10	16	7	2	1	21	3	11	1	6	0	1	13	0	1	11	11	0	2	4	2	1	3	2	4	10	0	9	4	0	18	10	13
pentylenetetrazol	95	278	133	70	41	50	60	3	1014	255	15	70	61	83	381	1	9	41	121	0	11	50	22	35	26	9	229	94	11	5	67	4	182	92	197
SNC80	77	1560	372	200	91	45	132	11	255	2308	34	188	44	60	1970	0	16	97	603	0	10	104	52	92	56	16	1408	206	20	6	153	3	1216	355	1179
strychnine	10	32	38	24	8	8	30	1	15	34	60	10	7	13	39	1	7	14	24	0	4	26	17	22	11	10	23	16	9	1	22	3	33	29	33
amitriptyline	41	172	98	96	42	26	46	6	70	188	10	346	17	22	220	0	9	48	151	0	2	47	28	27	39	9	176	85	10	6	68	0	109	110	125
aminophylline	72	54	38	18	12	59	23	0	61	44	7	17	158	49	58	2	5	18	29	0	4	14	9	9	10	5	45	25	7	0	19	2	51	18	41
bupropion	50	71	47	34	8	40	35	1	83	60	13	22	49	202	94	2	2	24	25	0	4	17	7	11	12	7	62	36	5	3	31	1	55	28	48
clozapine	128	2006	475	200	88	56	169	13	381	1970	39	220	58	94	3695	0	18	103	659	0	9	114	62	90	64	16	1730	257	24	10	165	4	1392	372	1295
diphenhydramine	1	1	2	2	2	1	2	0	1	0	1	0	2	2	0	3	1	0	2	0	0	1	1	1	2	3	0	3	1	1	3	0	2	2	2
isoniazid	4	15	7	17	4	2	7	1	9	16	7	9	5	2	18	1	32	8	8	0	7	18	6	14	5	5	16	6	4	2	14	4	7	21	11
maprotiline	22	116	53	92	32	15	18	11	41	97	14	48	18	24	103	0	8	141	72	0	8	29	16	15	18	7	53	68	4	11	48	2	77	75	76
mirtazapine	45	555	274	148	78	36	93	11	121	603	24	151	29	25	659	2	8	72	827	0	4	73	40	46	49	14	474	184	18	6	113	2	472	227	508
paroxetine	0	0	0	0	0	0	0	0	0	0	0	0	0	0	0	0	0	0	0	0	0	0	0	0	0	0	0	0	0	0	0	0	0	0	0
temozolomide	6	11	3	8	4	4	3	2	11	10	4	2	4	4	9	0	7	8	4	0	21	10	1	6	3	4	7	5	2	1	6	4	7	7	5
theophylline	22	90	64	52	26	14	46	4	50	104	26	47	14	17	114	1	18	29	73	0	10	200	31	52	23	14	107	45	16	5	58	5	58	71	90
tramadol HCl	19	48	43	20	20	10	32	2	22	52	17	28	9	7	62	1	6	16	40	0	1	31	100	22	16	10	56	26	12	2	25	1	42	37	57
venlafaxine HCl	9	71	39	33	10	5	34	1	35	92	22	27	9	11	90	1	14	15	46	0	6	52	22	151	14	7	75	20	12	1	38	3	34	60	63
azelastine	10	48	44	26	25	14	23	3	26	56	11	39	10	12	64	2	5	18	49	0	3	23	16	14	84	11	37	38	10	4	28	1	42	37	47
darifenacin	5	12	15	11	14	7	10	2	9	16	10	9	5	7	16	3	5	7	14	0	4	14	10	7	11	23	11	12	6	2	14	3	12	10	13
imiquimod	92	1190	297	114	57	46	118	4	229	1408	23	176	45	62	1730	0	16	53	474	0	7	107	56	75	37	11	2214	124	25	4	123	3	956	242	930
miconazole	34	232	130	113	66	30	43	10	94	206	16	85	25	36	257	3	6	68	184	0	5	45	26	20	38	12	124	332	10	9	80	2	185	125	196
minoxidil	4	19	16	10	7	7	13	0	11	20	9	10	7	5	24	1	4	4	18	0	2	16	12	12	10	6	25	10	36	1	8	2	14	12	18
niacin	0	12	8	14	7	2	3	9	5	6	1	6	0	3	10	1	2	11	6	0	1	5	2	1	4	2	4	9	1	15	7	0	12	9	8
ospemifene	21	135	93	77	25	22	48	4	67	153	22	68	19	31	165	3	14	48	113	0	6	58	25	38	28	14	123	80	8	7	259	3	105	116	138
roflumilast	1	3	2	4	2	1	2	0	4	3	3	0	2	1	4	0	4	2	2	0	4	5	1	3	1	3	3	2	2	0	3	7	3	4	2
rosiglitazone	81	1274	371	147	77	39	120	18	182	1216	33	109	51	55	1392	2	7	77	472	0	7	58	42	34	42	12	956	185	14	12	105	3	1827	236	1267
valdecoxib	21	315	153	133	51	22	51	10	92	355	29	110	18	28	372	2	21	75	227	0	7	71	37	60	37	10	242	125	12	9	116	4	236	479	280
DMSO	79	1173	387	149	78	40	137	13	197	1179	33	125	41	48	1295	2	11	76	508	0	5	90	57	63	47	13	930	196	18	8	138	2	1267	280	1686

**Table 4:** One-vs.-rest classification performance per compound group. Task denotes the compound groups attempted to separate and classifier denotes the ML model utilized in the classification. While tool compounds can be separated moderately well from the FAERS-positive (+) and FAERS-negative (−) compounds, the poor classification performance for the remaining groups implies the lack of a clear pattern distinguishing the FAERS− and FAERS+ compounds.

Task	Classifier	Accuracy	Sensitivity	Specificity	AUC
Tool vs. FAER +/−	*SVM*	*0.76*	*0.61*	*0.83*	*0.76*
	*LR*	*0.79*	*0.64*	*0.87*	*0.78*
FAERS+ vs. tool/FAERS−	*SVM*	*0.54*	*0.42*	*0.62*	*0.55*
	*LR*	*0.49*	*0.42*	*0.59*	*0.53*
FAERS− vs. tool/FAERS+	*SVM*	*0.59*	*0.31*	*0.77*	*0.59*
	*LR*	*0.72*	*0.49*	*0.76*	*0.72*
FAERS+ vs. FAERS−	*SVM*	*0.43*	*0.57*	*0.25*	*0.41*
	*LR*	*0.43*	*0.50*	*0.33*	*0.39*

**Table 5:** Alikeness-% between the tool and FAERS-positive compounds.

Tool Compounds (11)	FAERS-Positive Compounds (13)												
	Amitriptyline	Aminophylline	Bupropion	Clozapine	Diphenhydramine	Isoniazid	Maprotiline	Mirtazapine	Paroxetine	Temozolomide	Theophylline	Tramadol	Venlafaxine Hydrochloride
4-aminopyridine	2.9	18.4	9.8	9.8	0	0	1.1	1.7	0	0.6	1.7	0.6	0
amoxapine	2.1	0.3	0.8	50.2	0	0.1	1.5	4.3	0	0.1	0.9	0.2	1.0
bicuculline	4.7	2.0	2.0	28	0	0	2.0	17.3	0	0	1.3	1.3	0.7
chlorpromazine	16.4	0	4.9	29.5	0	0	18.0	11.5	0	3.3	6.6	1.6	1.6
donepezil	0	0	9.1	18.2	0	0	0	9.1	0	0	4.5	4.5	0
kainic acid	0	36.5	17.6	4.7	0	0	0	1.2	0	0	0	0	0
picrotoxin	3.5	0	1.2	11.8	0	0	1.2	9.4	0	0	5.9	2.4	1.2
pilocarpine HCl	0	0	0	0	0	0	0	0	0	0	0	0	0
pentylenetetrazol	1.1	4.4	5.2	7.4	0	0.2	0.3	1.1	0	0.3	0.2	0	0.5
SNC80	3.3	0.5	1.9	61.9	0	0	1.7	11.1	0	0.5	2.1	0.7	2.4
strychnine	0	0	0	0	0	0	12.5	0	0	12.5	12.5	0	0
**alikeness-% (sum)**	**34**	**62.1**	**52.5**	**221.5**	**0**	**0.3**	**38.3**	**66.7**	**0**	**17.3**	**35.7**	**11.3**	**7.4**

**Table 6:** Sum of the normalized enrichment score (NES) base values for the FEARS-positive compounds (GSEA score) and the number of enriched gene sets in the enrichment analysis with FEARS-positive drugs and tool compounds. For each tool compound, a gene set was generated from the upregulated and downregulated genes, which were then compared to a ranked list of each FEARS-positive compound.

FAERS-Positive Compound	Number of Significant Gene Sets in Enrichment Analysis	Sum of Significant NES Scores (GSEA Score)	Upregulated Genes Similar to Tool Compounds	Downregulated Genes Similar to Tool Compounds
clozapine	14	28.8	amoxapine bicuculline chlorpromazine donepezil pentylenetetrazol picrotoxin SNC80 strychnine	bicuculline chlorpromazine donepezil picrotoxin SNC80 strychnine
mirtazapine	11	20.1	4-aminopyridine amoxapine bicuculline chlorpromazine donepezil picrotoxin SNC80	amoxapine bicuculline chlorpromazine SNC80
bupropion	7	11.9	4-aminopyridine chlorpromazine donepezil kainic acid pentylenetetrazol	4-aminopyridine kainic acid
amitriptyline	5	8.5	chlorpromazine donepezil	amoxapine chlorpromazine SNC80
aminophylline	4	7.8	-	4-aminopyridine kainic acid pentylenetetrazol pilocarpine HCl
theophylline	3	5.1	-	amoxapine SNC80 strychnine
maprotiline	3	4.9	chlorpromazine	chlorpromazine SNC80
venlafaxine HCl	2	3.4	-	SNC80 strychnine

**Table 7:** Summary of correct categorization of FAERS-positive compounds by different analysis approaches. Diphenhydramine and paroxetine were excluded from the summary due to the low number of differentially expressed (DE) genes. The higher the rank, the greater the alikeness-% or GSEA score.

FAERS-Positive (11 Left)	Nr of DE Genes	Alikeness-%		GSEA Score		Machine Learning
		Correct Categorization	Rank	Correct Categorization	Rank	
amitriptyline	346	Yes	7	Yes	4	Yes
aminophylline	158	Yes	3	Yes	5	Yes
bupropion	202	Yes	4	Yes	3	Yes
clozapine	3 695	Yes	1	Yes	1	Yes
isoniazid	32	No		No		Yes
maprotiline	141	Yes	5	Yes	7	Yes
mirtazapine	827	Yes	2	Yes	2	Yes
temozolomide	21	Yes	8	No		Yes
theophylline	200	Yes	6	Yes	6	Yes
tramadol	100	Yes	9	No		Yes
venlafaxine hydrochloride	151	No		Yes	8	No
		9/11 (85%)		8/11 (73%)		10/11 (91%)

Abbreviations: DE, differentially expressed gene; FAERS, FDA Adverse Event Reporting System; GSEA, Gene Set Enrichment Analysis; Nr, number.

**Table 8:** Concentration range assessed (µM), compound-induced cytotoxicity in the rat cortical neuronal cultures, the highest tolerated or tested concentration, and the concentration selected for the compound-induced gene expression experiment of the 11 tool, 13 FAERS-positive, and 10 FAERS-negative compounds. The vendor and product number of the compounds purchased are shown in the right-hand columns. The concentrations for cytotoxicity testing were selected based on a literature review of known therapeutic plasma concentration in humans.

Type	Compound	Concentrations Tested (µM)	Cytotoxicity (µM)	Highest Tolerated/ Tested Concentration (µM)	Compound- Induced Gene Expression Experiment (µM)	Vendor	Product Number
**Tool**	4-aminopyridine	0.1; 1; 10; 100	none	100	100	Sigma	275875
**compounds**	amoxapine	0.1; 1; 10; 30	none	30	30	Sigma	A129
**(11)**	bicuculline	1; 10; 30; 100	none	100	100	Sigma	14340
	chlorpromazine hydrochloride	0.1;1;10;100	100	10	10	Sigma	31679
	donepezil	0.01; 0.1; 1; 10	none	10	10	Sigma	D6821
	kainic acid	0.1; 1; 10; 100	100	10	10	Sigma	420318
	pentylenetetrazol	100; 1000; 2500; 5000; 10,000; 20,000	none	20,000	20,000	Sigma	P6500
	picrotoxin	0.1; 1; 10; 30	none	30	30	Sigma	P1675
	pilocarpine hydrochloride	0.01; 0.1; 1; 10	none	10	10	Sigma	P6503
	SNC80	0.1; 1; 10; 30	none	30	30	Sigma	S2812
	strychnine hydrochloride	0.01; 0.1; 1; 10	none	10	10	Sigma	S8753
**FAERS-**	aminophylline	0.1; 1; 10; 100	none	100	100	Sigma	A1755
**positive**	amitriptyline hydrochloride	0.01; 0.1; 1; 10	none	10	10	Sigma	A8404
**compounds**	bupropion hydrochloride	0.1; 1; 10; 100	none	100	100	Sigma	B102
**(13)**	clozapine	0.1; 1; 10; 30	none	30	30	Sigma	C6305
	diphenhydramine hydrochloride	0.01; 0.1; 1; 10	none	10	10	Sigma	D3630
	isoniazid	1; 10; 30; 100	none	100	100	Sigma	I3377
	maprotiline hydrochloride	0.1; 1; 10; 30	30	10	10	Sigma	M9651
	mirtazapine	0.1; 1; 10; 30	none	30	30	Sigma	M0443
	paroxetine hydrochloride hemihydrate	0.01; 0.1; 1; 10	none	10	10	Sigma	P9623
	temozolomide	1; 10; 30; 100	100	30	30	Sigma	T2577
	theophylline	10; 30; 100; 1000	1000 *	100	100	Sigma	T1633
	tramadol hydrochloride	0.1; 1; 10; 30	none	30	30	Sigma	42965
	venlafaxine hydrochloride	0.01; 0.1; 1; 10	none	10	10	Sigma	V7264
**FAERS-**	azelastine hydrochloride	0.01; 0.1; 1; 10	none	10	10	Sigma	A7611
**negative**	darifenacin hydrobromide	0.001; 0.01; 0.1; 1	none	1	1	Sigma	SML1102
**compounds**	imiquimod	0.001; 0.01; 0.1; 1	none	1	1	Sigma	401020
**(10)**	miconazole	1; 10; 30; 100	30 and 100	10	10	Sigma	1443409
	minoxidil	0.1; 1; 10; 30	none	30	30	Sigma	M4145
	niacin	0.1; 1; 10; 30	none	30	30	Sigma	N4126
	ospemifene	0.1; 1; 10; 30	none	30	30	Sigma	SML0996
	roflumilast	0.01; 0.1; 1; 10	none	10	10	Sigma	SML1099
	rosiglitazone	1; 10; 30; 100	none	100	100	Sigma	R2408
	valdecoxib	0.1; 1; 10; 30	none	30	30	Sigma	PZ0179

* Statistical analysis indicated that a 1000 µM concentration of theophylline showed a trend towards cytotoxicity. Consequently, a lower concentration was used in the mRNA-seq experiment.

## Data Availability

The RNA-Seq data in FASTQ-files generated in this publication have been deposited with links to BioProject accession number PRJNA913400 in the NCBI BioProject database (https://www.ncbi.nlm.nih.gov/bioproject/ accessed on 13 December 2022).

## Supplementary Materials

The following supporting information can be downloaded at: https://www.mdpi.com/article/10.3390/ijms24044116/s1.
